# Algal Polysaccharides as Therapeutic Agents for Atherosclerosis

**DOI:** 10.3389/fcvm.2018.00153

**Published:** 2018-10-26

**Authors:** Nikita P. Patil, Victoria Le, Andrew D. Sligar, Lei Mei, Daniel Chavarria, Emily Y. Yang, Aaron B. Baker

**Affiliations:** ^1^Department of Biomedical Engineering, University of Texas at Austin, Austin, TX, United States; ^2^Institute for Cellular and Molecular Biology, University of Texas at Austin, Austin, TX, United States; ^3^Institute for Computational Engineering and Sciences, University of Texas at Austin, Austin, TX, United States; ^4^Institute for Biomaterials, Drug Delivery and Regenerative Medicine, University of Texas at Austin, Austin, TX, United States

**Keywords:** atherosclerosis, seaweed, fucoidan, ulvan, laminarin, alginate, hyperlipidemia, microbiome

## Abstract

Seaweed-derived polysaccharides including agar and alginate, have found widespread applications in biomedical research and medical therapeutic applications including wound healing, drug delivery, and tissue engineering. Given the recent increases in the incidence of diabetes, obesity and hyperlipidemia, there is a pressing need for low cost therapeutics that can economically and effectively slow the progression of atherosclerosis. Marine polysaccharides have been consumed by humans for millennia and are available in large quantities at low cost. Polysaccharides such as fucoidan, laminarin sulfate and ulvan have shown promise in reducing atherosclerosis and its accompanying risk factors in animal models. However, others have been tested in very limited context in scientific studies. In this review, we explore the current state of knowledge for these promising therapeutics and discuss the potential and challenges of using seaweed derived polysaccharides as therapies for atherosclerosis.

## Introduction

In recent times, there has been an increase in the global incidence of risk factors for atherosclerosis including obesity, hyperlipidemia and diabetes. The statins have become a ubiquitous part of the clinical treatment for atherosclerotic disease. However, many patients experience side effects with statins and withdrawal from these drugs may lead to increased cardiovascular or neurovascular events (1, 2). Consequently, there is great clinical need for therapies that can reduce the incidence and progression of atherosclerotic vascular disease beyond what is possible with current treatments. Polysaccharides derived from seaweeds are appealing therapeutics for atherosclerosis for their favorable economics, availability and low toxicity. In the 1970s, there was increasing interest in using seaweed-derived polysaccharides as lipid lowering drugs and inhibitors of atherosclerotic plaque formation. However, the discovery of statins during this time eclipsed many promising studies on other compounds, driving the field in the direction of statin-focused research for many years. With the recent realization that there can be significant side effects to long term statin therapy and that treatment with these compounds can only slow the progression of atherosclerosis, there has been increasing interest in exploring alternative or complementary therapies.

**Figure 1:**
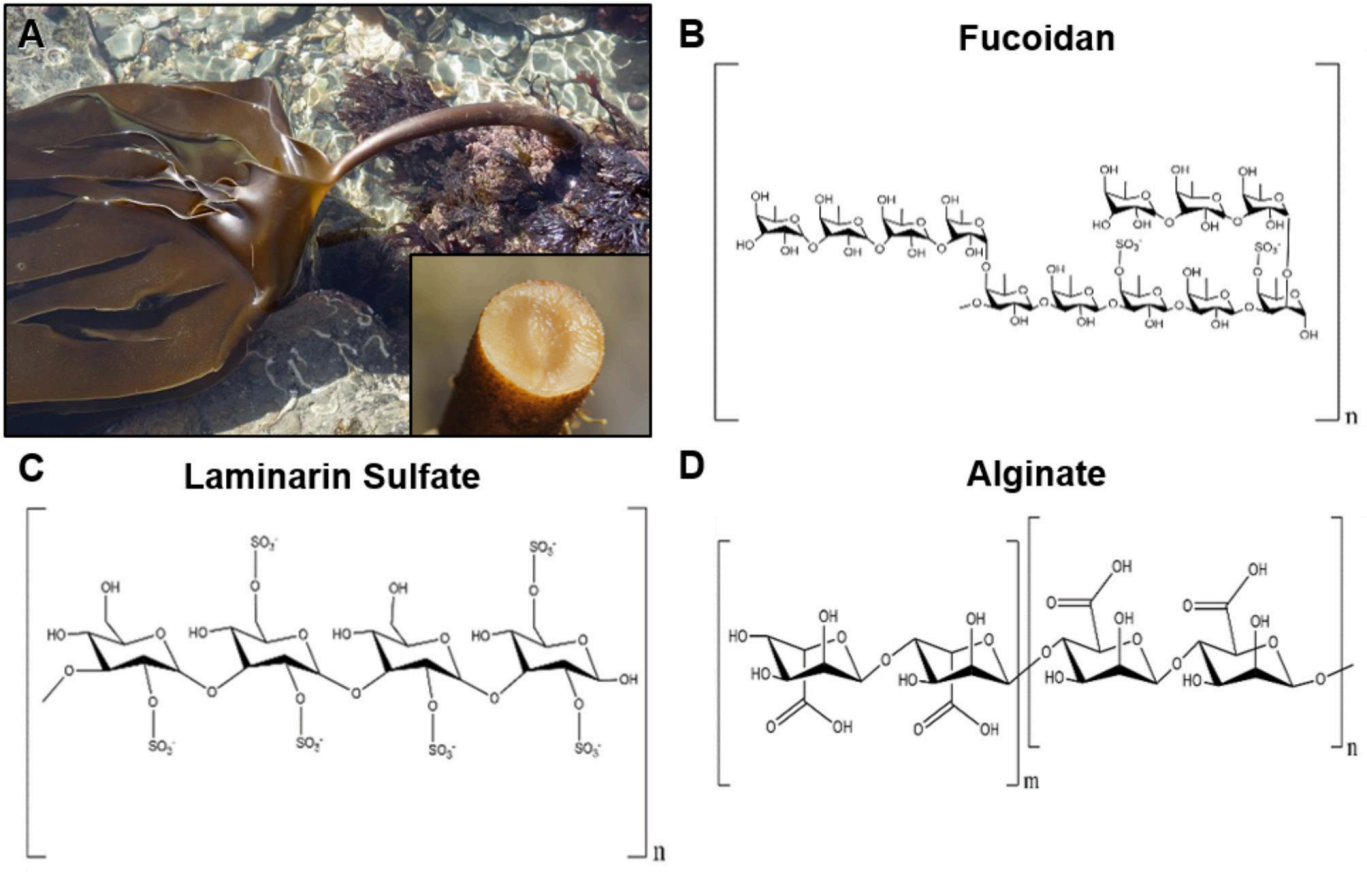
Representative image of brown seaweeds (*phaeophyceae*) and chemical structures of derived polysaccharides. **(A)** Photograph of the brown seaweed *laminaria digitata*, which is a commons source for the polysaccharide laminarin. The inset image shows the cross section of the holdfast from the brown seaweed *laminaria hyperborean*. Photographs courtesy of David Fenwick, used with permission^1^. **(B)** Chemical structure of fucoidan derived from brown seaweeds. **(C)** Structure of laminarin sulfate, a chemically modified version of laminarin that is commonly derived from seaweeds of the family *Laminariaceae*. **(D)** Chemical structure of alginate, which is derived from the cell wall of brown seaweeds.

**Figure 2:**
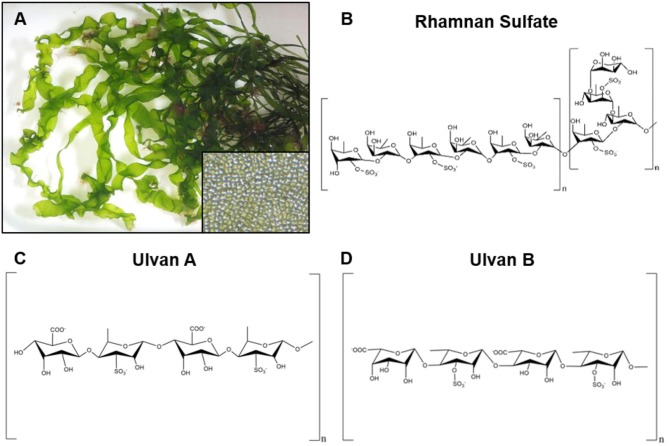
Representative image of red seaweeds (*rhodophyta*) and chemical structure of derived polysaccharides. **(A)** Photograph of the green seaweed *ulva linza*, a source of the polysaccharide ulvan. Inset image is a magnified view of the cellular structure of *ulva linza*. Photographs courtesy of David Fenwick, used with permission^1^. **(B)** Chemical structure of rhamnan sulfate, a branched polysaccharide derived from the green seaweeds *monostrom nitidum* and *monostroma latissimum*. **(C,D)** Two chemical structures for ulvan that have been isolated from green seaweeds.

**Figure 3:**
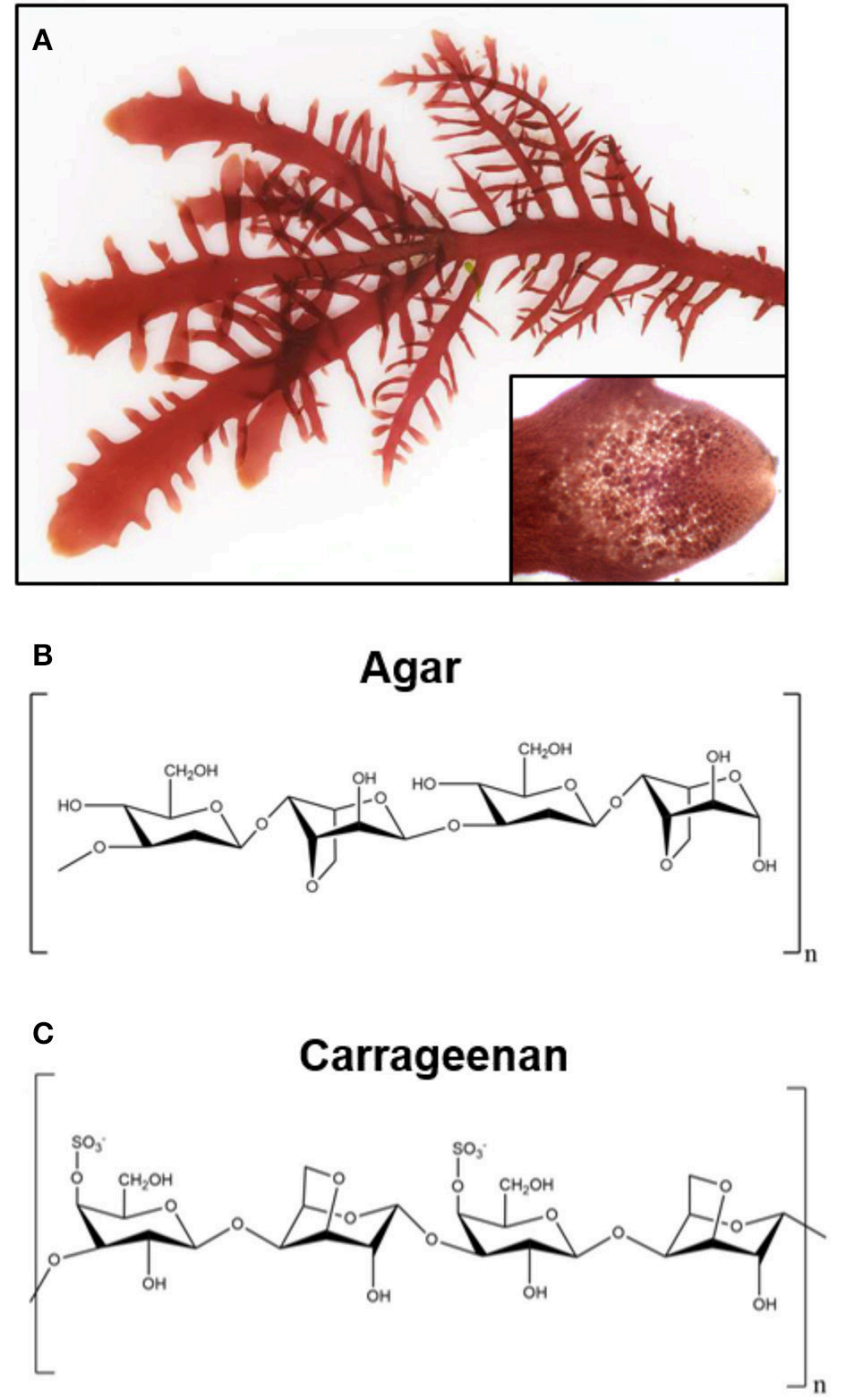
Representative image of green seaweeds (*chlorophyta/charophyta*) and chemical structure of derived polysaccharides. **(A)** Photograph of the red seaweed *gelidium pusillum*, a source of agar. Inset image is a magnified view of the cellular structure of the *gelidium pusillum* frond. Photographs courtesy of David Fenwick, used with permission^1^. **(B)** Chemical structure of polysaccharide agar, derived from red algae. **(C)** Generalized chemical structure of carrageenan, a linear polysaccharide from edible red seaweeds.

Polysaccharides derived from red, brown and green seaweeds have been identified to act on atherosclerosis or its risk factors. It is important to keep in mind that the structures of the polysaccharides obtained from seaweed depend on the species, geographic origin, time of harvest, and extraction method. In general, there is a high degree of heterogeneity in the size, sulfation and branching in many of the polysaccharides obtain from natural sources (3). While this seems a daunting aspect of these potential therapies, the commonly used anti-coagulant heparin has similar issues in its heterogeneity and variability with source. The structure, classification and manufacturing related issues have been discussed in several previous reviews and will not be reviewed extensively here (4–7). Figures 1–3 lists the major polysaccharides that have been explored for use as therapies for cardiovascular disease. Seaweeds are routinely harvested in large quantities for use as food, making them inexpensive to obtain worldwide. Historically, seaweeds have been consumed for centuries without any identifiable toxicity. Although some of their derivatives are used in food, research or other industrial applications, they still remain relatively unexplored as therapeutics.

This review will focus on the major algal polysaccharides in seaweeds that have shown potential for having direct or indirect effects on atherosclerosis. For each polysaccharide, we will^1^ discuss the structure of the active compound and its efficiency in atherosclerotic plaque reduction, if known. In addition, we will examine the potential for the polysaccharides for acting on risk factors for atherosclerosis including reduction of lipids, coagulation, oxidative stress, inflammation and the modulation of the microbiome.

## Fucoidan

Fucoidan is a polysaccharide composed of L-fucose that is derived from brown seaweed. Its chemical structure varies greatly based on a variety of factors including the species of seaweed and extraction methods (8). The most studied and commercially available form of fucoidan is prepared from *Fucus vesiculosus*, and is comprised of 44% fucose and 26% sulfate (9). Ordered fucoidans have been shown to contain a linear backbone of (1 → 3)-α-L-Fucose or alternating (1 → 3)- α-L-Fucose and (1 → 4)-α-L-Fucose, while sulfate groups often occupy the C-2, C-3, and C-4 of fucose (9).

**Figure 4:**
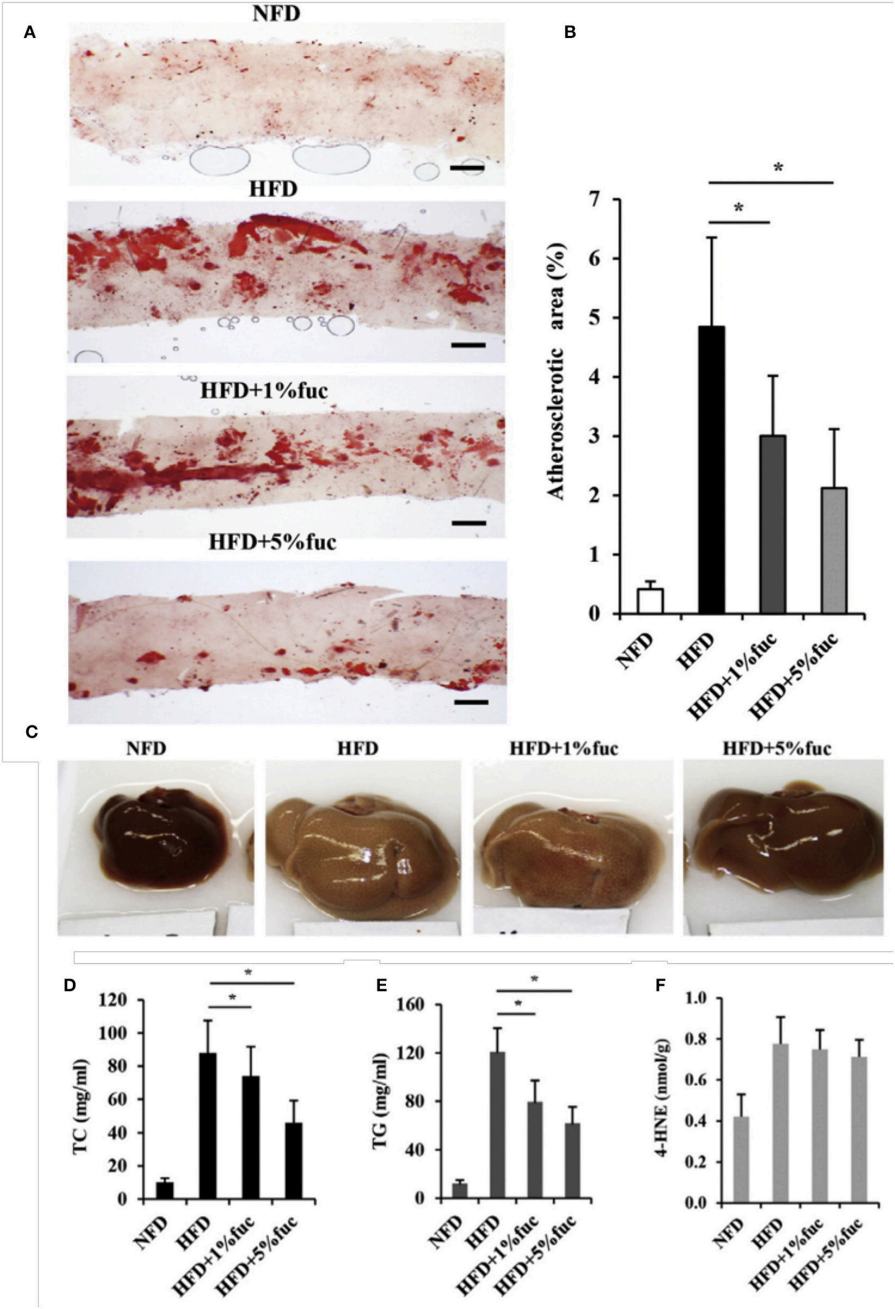
Effects of fucoidan on atherosclerosis and hepatic lipid metabolism. ApoE^*shl*^ mice were treated under control conditions (NFD), with a high fat diet (HFD), and a high fat diet with 1% and 5% fucoidan for 12 weeks (*n* = 6). **(A)** Oil-red O staining of intima of thoracic aorta to show atherosclerotic lesions. **(B)** Quantification of lesion area to total aorta calculated using ImageJ. The thickness of the intima was normalized to the thickness of the media to calculate the percentage of plaque. **(C)** Appearance of liver. **(D–F)** Total cholesterol, triacylglycerol and 4-hydroxynonenal concentrations in liver. Values are mean. ^*^p < 0.05 vs. HFD. Modified and used with permission (11).

Fucoidan is the most extensively studied marine polysaccharide in terms of its effects on atherosclerosis. A study in which low density lipoprotein receptor null (LDLR^−/−^) mice were fed a high cholesterol diet and treated with 100 mg/kg fucoidan through intragastric gavage, found that atherosclerotic plaques were significantly reduced in the aortic arch, descending thoracic aorta and abdominal aorta (10). In another study, spontaneously hyperlipidemic (Apo^*shl*^) mice were fed a high fat diet with fucoidan consisting of 1% and 5% of their daily food intake (11). After 12 weeks, the atherosclerotic lesion area of their aortas was significantly lower in the mice treated with fucoidan (Figures 4A,B) (11). These results were also supported by a study in which poloxamer-407 (P-407) was used to induce atherosclerotic lesions and hyperlipidemia in mice through lipid derangement (12, 13). Lesion formation in the aorta was significantly reduced by intraperitoneal administration of 50 mg/kg of fucoidan every third day for 16 weeks (12).

One potential mechanism of action for fucoidan's activity on atherosclerosis is the alteration in lipid uptake and metabolism. Total cholesterol, triglycerides and low density lipoprotein (LDL) levels in the serum were significantly reduced while high density lipoprotein (HDL) levels were increased starting from doses of 30 mg/kg in mice (10–12) and 100 mg/kg in rats (14–16). Due to fucoidan's additional effect on dyslipidemia, the liver has been identified as a possible target with research focusing on hepatic lipid metabolism. Peroxisome proliferator-activated receptor alpha (PPARα), which regulates hepatic fatty acid β-oxidation and is decreased in high fat diets, had significantly upregulated gene expression in the Apo^*shl*^ study described previously (11). Sterol regulatory element-binding protein 1 (SREBP1) regulates expression of lipogenic genes acetyl-CoA-carboxylase (ACC) and fatty acid synthetase (FAS). In the same study, SREBP1 was found to be downregulated by fucoidan treatment (Figures 4C–F) (11) but unchanged in human hepatoma cells and the P-407 mice, although ACC and FAS had decreased expression (12). The bile acid synthesis pathway, controlled by Cytochrome P450 Family 7 Subfamily A Member 1 (CYP7A1) and induced by SREBP1, was also downregulated after treatment with fucoidan (11). Sterol regulatory element-binding protein 2 (SREBP2), which regulates genes critical to cholesterol synthesis and uptake such as HMGCR and LDLR, was downregulated by fucoidan treatment in both human hepatoma cells and P-407 mice (12). These results suggest that fucoidan alters atherosclerotic plaque development by increasing lipid metabolism and decreasing lipid synthesis and uptake.

Fucoidan has also been shown to alter signaling through reactive oxidative species (ROS). In the previously described LDLR^−/−^ mice study, lectin-like oxidized LDL receptor-1 (LOX-1) along with reactive oxidative species (ROS) related proteins were downregulated in the aorta after fucoidan treatment (10). These results were supported by a study in streptozotocin (STZ) induced diabetic rats treated with 100 mg/kg/day fucoidan through intragastric administration for 12 weeks where ROS production was significantly decreased in the vascular smooth muscle cells of the aorta (14). Because ROS can modify LDL and LOX-1 is upregulated under pro-inflammatory conditions, their decreased expression under fucoidan treatment suggests that the marine polysaccharide reduces oxidative stress in atherosclerosis. While reduction of oxidative stress and inflammatory markers in the aorta could be a side effect of hepatic lipid metabolism, ROS have been known to cause a disruption in hepatic homeostasis (11). Consequently, there is not a consensus for the mechanism of fucoidan's action due to inconsistencies between reports. A study on the effect of fucoidan on oxidative stress in the liver and its influence on atherogenic markers could provide more insight on the mechanism behind fucoidan's reduction of atherosclerosis.

One of the first steps in the development of atherosclerotic plaques is the activation and adhesion of platelets to the endothelium (17). Selectins are integral in promoting platelet and leukocyte rolling and adhesion (18–20). Fucoidan acts as a ligand to both P- and L-selectin and inhibits their activity (19, 21). P-selectin, expressed on the surface of activated platelets and endothelial cells, promotes the adhesion of leukocytes that contribute to the growth of thrombi (22). *In vitro* studies showed fucoidan binding to P-selectin coated microtiter plates (23) and activated platelets (24). An *in vivo* study in STZ-induced diabetic rats found that daily oral administration of both 100 and 200 mg/kg of fucoidan reduced P-selectin expression in the glomerulus (25). Another study in rats given peritoneal inflammation through peptone injections indicated that fucoidan injected intravenously had the highest anti-inflammatory effect when administered at an early stage (15 min after peptone injection) when P-selectin expression is maximum (26). These *in vivo* results were supported by an *in vitro* assay where fucoidan showed P-selectin binding comparable to SiaLe^a/x^-PAA-biot, a P-selectin ligand (26). Also, fucoidan has been used as a P-selectin targeting agent in imaging aortas of ApoE^−/−^ mice (18) and arterial thrombi in rats with either abdominal aortic aneurysms or infective endocarditis (24).

L-selectin is constitutively expressed on the surface of leukocytes and neutrophils and mediates leukocyte rolling and neutrophil adhesion to inflamed endothelial cells (27). Fucoidan has long been established as an L-selectin inhibitor and is used as a blockade agent that can reduce inflammation (28, 29). An *in vivo* study in rat mesenteric arteries indicated that fucoidan treatment at 1 mg/mL reduced leukocyte rolling by 94% during 31 micro-infusion applications without affecting concentration of leukocytes (20). Another study examining the pulmonary microvessels of rabbits found that a 20 mg/kg IV injection of fucoidan reduced leukocyte rolling in arterioles and venules by 75 and 83% respectively and increased leukocyte flow velocity in capillaries by 51% (30). Fucoidan as a selectin inhibitor shows promise as an anti-inflammatory agent that could treat atherosclerosis at an early stage.

Fucoidan has also had efficacy in preventing diabetes in some animal studies. The STZ-induced diabetic rats described earlier had significantly elevated blood pressure and glucose levels along with lowered body weight (14). The 100 mg/kg dose of fucoidan reduced both systolic and diastolic blood pressures and protected against weight loss, but did not affect hyperglycemia (14). Goto-Kakizaki rats are a non-obese model of type 2 diabetes. When these animals were treated with fucoidan through intragastric gavage for 12 weeks there was a significant decrease in hypertension with 50, 100, and 200 mg/kg/day doses without changes in hyperglycemia or body weight (15). In a different study, NG-nitro-L-arginine methyl ester (L-NAME) was used to induce hypertension in mice. Oral administration of 100 mg/kg/day of fucoidan for 4 weeks in these animals decreased blood pressure and increased production of nitric oxide production (31). Notably, many of these *in vivo* studies were done in male rats and mice only and further research is needed to evaluate their efficacy in female animals.

Fucoidans exhibit anticoagulant activity that is largely dependent on the seaweed from which they are extracted (9, 29). For most fucoidans, increases in both activated partial thromboplastin time (aPTT) and thromboplastin time (TT) were observed, on a scale either comparable to or higher than heparin (9). Interestingly, the increase in clotting time from fucoidan treatment was higher in male rather than female Wistar rats (32). Also, some fucoidans showed anti-thrombin activity on the order of heparin (29). In general, anticoagulation from fucoidan appeared to depend on a higher sulfate content, increased molecular weight and position of sulfate groups on the backbone (9, 33). Hence, fucoidan has the potential to be a plant-based alternative to heparin (34).

Despite the observed reduction of atherosclerotic plaques after treatment with fucoidan, there is evidence of the polysaccharide contributing to macrophage apoptosis, a stage crucial to the formation of necrotic cores in the plaques. An *in vitro* study in murine macrophages showed that fucoidan increased the secretion of tumor necrosis factor-alpha (TNF-α), due to its ability to bind macrophage scavenger receptor class A (SR-A) (35). This ligand-receptor interaction has been used to study the pathways involved in macrophage apoptosis. Separate *in vitro* studies in murine macrophages showed that fucoidan binding to SR-A activates the p38 mitogen-activated protein kinase (MAPK) and c-Jun N-terminal kinases (JNKs) (35–37). However, both endoplasmic reticular stress and the SR-A pathway are required to cause macrophage death, hence fucoidan alone could not induce apoptosis to a significant degree (35–37). This non-specific binding to SR-A is especially relevant to the later stages of atherosclerosis in humans, when the plaques rupture. Because mouse and rat models lack this developmental stage, a study in higher order animals with fucoidan may be a better indicator of the therapeutic activity.

There has also been some research done on the effect of fucoidan on gut bacteria and its correlation in alleviating risk factors of many diseases including atherosclerosis. *In vitro* studies on human fecal samples showed that treatment with the brown seaweed *Ascophyllum nodosum*, which is rich in fucoidan content, led to increased ratio of *Bacteroidetes* to *Firmicutes* (38). An increase in this ratio is associated with reduced bacterial energy use and reduced risk of obesity (38). There was also an increase in short chain fatty acids (SCFAs) along with higher growth of *Ruminococcaceae*, SCFA producers, in multiple *in vitro* and *in vivo* studies in pigs whose diets had been supplemented with fucoidan (38–41). The most profuse SCFAs are acetic acid, which prevents enteropathogenic activity, and propionic acid, which influences cholesterol metabolism (38). Also, a study in C57BL/6 mice fed a high fat diet showed that the activity of the microbial enzyme bile salt hydrolase was reduced in mice whose diets were supplemented with brown algae extract that would lead to decreased lipid absorption (42). Further, research is necessary to study the direct effects of these microbial changes to atherosclerosis progression, however, these studies present an additional aspect of fucoidan's anti-atherogenic potential.

## Laminarin sulfate

**Figure 5:**
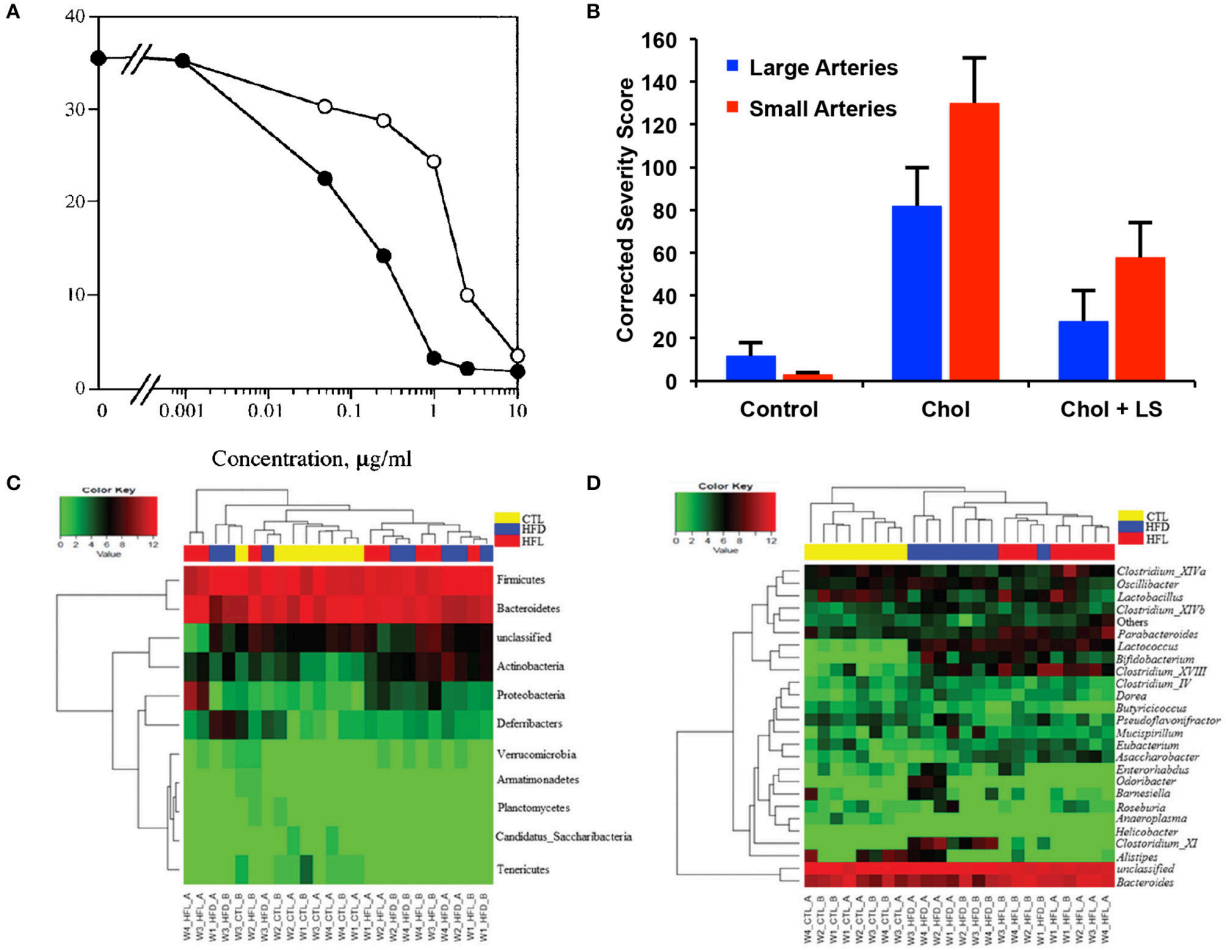
Effects of laminarin sulfate on atherosclerosis, heparanase and the intestinal microbiome. **(A)** Inhibition of heparanase by Inhibition of purified placental heparanase. Radiolabeled ECM was incubated with heparanase in the presence of LS (•) or heparin (○). Heparanase activity is expressed as K_av_ x cpm eluted in the second peak when the incubation media was analyzed by gel filtration chromatography. Modified and used with permission (47). **(B)** Severity of plaques in the small and large coronary arteries of rabbits that were treated under control conditions, with a high cholesterol diet (chol) or a high cholesterol diet with laminarin sulfate (LS). Male New Zealand rabbits were used with *n* = 26 for the control group, *n* = 24 for the chol group and *n* = 25 for the chol + LS group. Scores have been adjusted with maximum possible being 370 for large arteries and 544 for small arteries. Modified and used with permission (48). (C) Abundance of phylum **(A)** and genus levels **(B)** of bacteria in the gut in mice fed a high fat diet and laminarin sulfate for 42 days. Modified and used with permission (49).

Laminarin is a polysaccharide composed of D-glucopyranose connected through 1, 3 β-glycosidic linkages with average length of 20–25 disaccharides (43). It is found in some brown algae and in high concentrations in members of the genus *laminaria* (kelp) where it composes 30–50% of the dry weight (44) as well as other species of Laminariales and Fucales (45). Laminarin can be chemically sulfated, which increases its biological activity in many cases (46). Laminarin sulfate reduces metastasis in experimental models through inhibition of heparanase, an endo-β-glucuronidase that cleaves heparan sulfate (Figure 5A) (47). Heparanase has been linked to multiple pro-atherogenic processes including restenosis following vascular injury and stenting (50), thrombosis (51), and has been associated with vulnerable plaque morphology in a diabetic, hyperlipidemic porcine model of atherosclerosis (52). In mice, increased expression of heparanase enhances fatty streak formation in the arteries (53). A study of 127 human tissue samples harvested during endarterectomies of the carotid artery found that there was increased amounts of heparanase in arteries with plaque vs. normal arterial tissues (54). In addition, patients with symptomatic lesions had increase heparanase over those with plaque. These findings were consistent with another study that showed increased plasma heparanase levels in patients with myocardial infarction and in vulnerable plaques in the coronary arteries (55). Heparanase has also been linked to shedding of the glycocalyx in septic shock (56). Loss of heparan sulfate due to heparanase is known to enhance shedding of cell surface proteoglycans including syndecan-1 (57, 58). Further, a reduction in syndecan-1 in endothelial cells leads to altered mechanotransduction and a pro-inflammatory phenotype (59). Thus, reduction of heparanase activity by laminarin may be able to reduce atherogenesis and continued progression of atherosclerotic plaques. In addition to its inhibitory activity toward heparanase, laminarin sulfate also has other activities that potentially could be useful in treating atherosclerosis. Laminarin sulfate binds to the heparin-binding region of fibroblast growth factor 2 (FGF-2) and inhibits the binding of FGF-2 to its receptor (60, 61). Moreover, addition of laminarin sulfate dissociates FGF-2 from the low binding affinity sites on the cell. While FGF-2 activity may be beneficial by providing an angiogenic stimulus in regions of tissue made ischemic by atherosclerotic disease, it also has a role in driving the processes involved in progression of atherosclerotic plaques (62).

Like other marine polysaccharides, laminarin sulfate has been found to have lipid lowering (48, 63, 64) and anti-coagulant properties (64). In a rabbit model of atherosclerosis with intermittent cholesterol feeding, laminarin sulfate treatment induced a dramatic reduction in plaque grade and intimal cholesterol (Figure 5B) (48). Notably, the benefits of laminarin sulfate appeared to be greater for male rabbits in comparison to female rabbits undergoing the same treatments. Laminarin has been shown to work as an antioxidant and free radical scavenger with an EC_50_ (dose for 50% free radical neutralization) of 460 μg/ml (65). The mechanism for the free radical scavenging was hypothesized to be the abstraction of the anomeric hydrogen within polysaccharides, theoretically accounting for the improved antioxidant activity of polysaccharides over monosaccharides. Gamma irradiation in the range of 0–200 kGy induced a dose-dependent increase in the ferric reducing antioxidant potential of laminarin, corresponding with enhanced free radical scavenging activity (66). This irradiation also increased the amount of reducing sugars and decreased in molecular weight of the laminarin.

Laminarin may also affect immunomodulatory pathways that are associated with atherosclerotic progression or risk factors for atherosclerosis. Laminarin can be either an agonist or an antagonist toward dectin-1 depending on its physical properties including the purity and molecular weight (67). Dectin-1 is a pattern recognition receptor that plays in a role in the activation of antifungal innate immunity. While dectin-1 knockout in the bone marrow of LDLR^−/−^ mice did not alter the progression of atherosclerosis (68), a recent study found that dectin-1 signaling enhanced obesity and insulin resistance in mice with in MyD88 knockout mice fed a high fat diet (69). In humans, expression of MyD88 has been associated with obesity and metabolic syndrome (70). Dietary supplementation with laminarin-reduced hepatotoxicity in rats treated with lipopolysaccharide (LPS), suggesting an anti-inflammatory effect for some formulations of laminarin (71).

Laminarin has also been shown to modify the bacteria in the gut, providing potential benefits to preventing or reducing atherosclerosis. In mice fed a high fat diet, laminarin ingestion correlated with an increase in bacteria that are associated with gut health including *Bacteroides, Parabacteroides* and *Clostridium* cluster XIVa (Figures 5C,D) (49). This shift in gut flora also led to alterations in the levels of carbohydrate-active enzymes (CAZymes), including an increase in enzymes associated with reduced body mass index in humans. These shifts in bacteria in the gut reversed with cessation of laminarin feeding, suggesting continued consumption of the compound would be needed to counteract the effects of a high fat diet. In pigs, feeding with laminarin led to a reduction in *Enterobacterium* spp. in the colon while a combination of fucoidan and laminarin led increased colonic *Enterobacterium* (72). A metagenomic association study found that patients with atherosclerotic cardiovascular disease had increased amounts of *Enterobacteriaceae* and *Streptococcus* spp., suggesting that marine polysaccharides may have the potential to shift the gut microbiome to reduce the impact of a high fat diet on atherosclerosis.

## Alginate

Alginate is a naturally originating polysaccharide from brown seaweed and has been used extensively in the biotechnology industry. For many years in the food and beverage industry, alginate has been used as a thickening agent, gelating agent and a colloidal stabilizer (73). In biomedical science, alginate is also extensively researched as a biomaterial due to its biocompatibility, with low toxicity and convenient availability. Alginate can be obtained from both algae and bacteria; however, commercially available sodium and calcium alginate is only extracted from seaweed *Phaeophyceae* by treatment with alkali, followed by filtration and precipitation by adding sodium or calcium chloride. Water-soluble sodium alginate powder can be produced by further treatment with dilute HCl, purification and dessication (74). In polymer structure, alginates are linear block copolymers composed of 1,4-linked β-D-mannuronic acid (M) with ^4^C_1_ ring conformation and α-L-guluronic acid (G) with ^4^C_1_ conformation, both in the pyranosic conformation and present in varying amounts (74). Haug et al. showed that alginates are formed of three types of blocks: block of GGGGG; block of MMMMM; and blocks of alternating M and G residues (75, 76). Moreover, the polymer composition as M/G ratio, especially G-block length, plays a key role in determining the physicochemical properties of alginate (77).

Alginate fibers are commonly used as thickener and stabilizer in the food industry. However, the physiological effects of alginates in animal and human bodies still remain unclear. In the recent decades, more research interest has been focused on investigating whether alginate fibers can lower cholesterol levels, LDL levels and HDL levels. Substantial *in vivo* studies have shown that various forms of alginates lead to hypocholesterolemic and hypolipidemic responses. Mushollaeni group recently indicated that the alginates extracted from brown seaweed *Sargassum duplicatum* and *Turbinaria* sp. that were derived from the beaches in Yogyakarta had the ability to decrease total blood cholesterol levels in pretreated hypercholesterolemic rats (78). In another study, alginate was added to a cholesterol-rich diet to rats for 2 weeks and the effects of alginate on ingestion and excretion of cholesterol *in vivo* were examined. It was found that alginate supplemented diets increased the efficiency of food digestion as well as decreasing the cholesterol level in liver (79). Furthermore, Amano et al. reported that a seaweed mixture of 95% brown seaweed and 5% red seaweed given in the diet for 28 days decreased serum total cholesterol, LDL-cholesterol, free cholesterol, and triglyceride levels significantly in the rats that were fed a cholesterol-rich diet compared to the control that received no seaweed in diet (80). Kartika group examined that daily feeding sodium alginates of either 2% liquid form or 0.02 mg/kg powder form could inhibit the formation of foam cells in high cholesterol feed rats, as well as lowering serum LDLs and increasing HDLs. After 12-week treatment, the observed foam cell numbers in alginates supplemented diet group decreased to about 30% and the body weight, cholesterol levels, LDL levels, HDL levels and triglyceride levels remained very close to the regular diet fed group (81).

Alginate can be modified into various derivatives and the most common modifications include acetylation, phosphorylation and sulfation. Sulfation of polysaccharides can render the blood compatibility and anticoagulant activity (82). Therefore, growing attention has been attracted to investigate if alginate sulfates have similar anticoagulation properties. Huang et al. first reported alginate sulfate had considerably high anticoagulant activity especially to the intrinsic coagulation pathway. In the study, the aPTT, TT, and prothrombin time (PT) were measured and it was showed that alginate sulfate greatly prolonged aPTT, but seldom influenced TT and PT (83). Xin et al. injected propylene glycol alginate sodium sulfate (PSS) intraperitoneally on mice and employed the glass capillary tube rupture test to evaluate hemorrhagic effects and clotting time. It was demonstrated that PSS significantly increased clotting and bleeding time, as well as decreasing the wet weights and lengths of the thrombus *in vivo* (84).

Besides its anticoagulant bioactivity, Zhao group isolated low-molecular-weight polyguluronate sulfate (LPGS) from alginate and administered LPGS at dose-levels ranging from 12.5 to 100 mg/kg on rats, followed by subcutaneously implanting sterile cotton pellets to induce inflammation and granuloma. It was showed that LPGS of all doses exhibited considerable anti-inflammatory activity in rats by reducing both the wet and dry weights of the granuloma (85). As coagulation and inflammation are significantly involved in the cause and progression of cardiovascular diseases, more evaluations on anti-inflammatory and anticoagulant properties of alginate are worthy to be done in the field. In the past decade, more studies on alginate also focused on the other biological activities with potential health benefits. For example, alginate has been examined to show antioxidant activity by ferric reducing antioxidant power (FRAP) and 2,2-diphenyl-1-picrylhydrazyl (DPPH) assays (86). However, its potential effect in hypertension, gut bacteria/probiotic activity along with other bioactive roles remains to be further explored.

## Rhamnan sulfate

Rhamnan sulfate is a major component of the green seaweed Monostroma Nitidum that has been less studies than other marine polysaccharides. It is primarily composed of L-rhamnose sugars linked through the α-1,3 carbons (87). In an early paper Harada and Maeda isolated and characterized rhamnan sulfate and demonstrated some anticoagulant properties (87). Since then, limited work has been done with the compound and its applications in cardiovascular diseases, but its anticoagulant activity has been studied to some extent. A low molecular weight form was shown to have higher anticoagulant activity than heparin at high concentrations as determined by the aPTT assay (88). The TT activity was also increased in higher concentrations of rhamnan sulfate indicating an effect on fibrin polymerization (89). Multiple studies also showed that the thrombin inhibition was carried out through the heparin cofactor II-dependent pathway (88, 89).

## Ulvan

Ulvan is a sulfated heteropolysacharide that is composed of disaccharide units of D-glucuronic acid or L-iduronic acid attached through a sulfated 1,4 L-rhamnose (90) residue with traces of D-xylose and D-glucose (91). The chemical composition of ulvan can vary due to a variety of reasons including the species it is extracted from, geographical location and harvest. The marine polysaccharide is found in green algae and is commonly extracted from *Ulva pertusa* (92) and *Ulva lactuca* (93). Green alga is consumed throughout Asian countries (92) and has been utilized in traditional Chinese medicine (94). France was the first European country to approve ulvan consumption due to its high vitamin and fiber content but it has quickly spread throughout the European region (95). The nutritional benefits of ulvan have been recognized around the world but its benefits as a therapeutic remain unknown.

There is potential for ulvan as a therapeutic to combat atherosclerosis. The direct effects of ulvan on atherosclerosis have not been fully explored but there is promise as it can reduce the atherogenic index (AI) of male hypercholesterolemic Wister rats by 95% with ulvan derived from *Ulva fasciata* when fed *ad libitum* for 4 weeks (96). Ulvan has been shown to reduce triglycerides (TG), total cholesterol (TC), and total lipid levels by 46, 69, and 30%, respectively, in hypercholesterolemic rats (96). This extraction method also significantly decreased low density lipoprotein cholesterol (LDL-C) and very low-density lipoprotein cholesterol (VLDL-C), respectively (96). Ulvan derivatives with higher concentrations of sulfate exert an increased antihyperlipidemic and antioxidant effect compared to natural ulvan (97). Acetylated ulvan administered at a concentration of 125 and 500 mg/kg led to a significant decrease in TC and LDL-C levels when compared to the hyperlipidemic Kunming mice control group (92). Few side effects have been noted from the consumption of ulvan. One side effect noted by Yu et al. and collaborators was an increase in bile acid production when rats were fed the polysaccharide from *Ulva pertusa* (98). It has been widely established that several forms of ulvan possess a lipid lowering effect that reduces TG, TC, LDL-C, VLDL-C, and increases HDL-C levels in both mice (92, 99) and rats (96, 97, 100).

Ulvan can be chemically modified to attach functional groups to the polysaccharide and improve its antioxidant abilities. Acetylated and benzoylated ulvan exhibited higher antioxidant effects compared to natural ulvan (101). Acetylated ulvan presented the strongest *in vitro* chelating ability in the presence of iron compared to benzoylated ulvan and ulvan, (102) suggesting that the chelating ability of these ulvan complexes can serve as effective antioxidants by reducing the redox potential of oxidized metal ions. High sulfate content (98) and molecular weight (93) ulvan led to increased antioxidant activity. Sulfate content seems to be especially important in the antioxidant effect of ulvan. In hyperlipidemic rats at a concentration of 125 mg/kg of high sulfate content ulvan led to the inhibition of malondialdehyde, a key player in lipid peroxidation, and increased activity of superoxide dismutase and catalase (98). This upregulation in the activity of radical scavenging proteins contributes to the antioxidant properties of ulvan *in vivo*. A positive linear correlation exists between the sulfate content of ulvan and its antioxidant activity in a DPPH assay (93). However, an increase in molecular weight may also lead to a decrease in the antihyperlipidemic effects of ulvan (97). Further research is needed to determine the optimal molecular weight of ulvan to maximize both the antioxidant and antihyperlipidemic effects of this marine polysaccharide.

## Agar

Agar is a linear polysaccharide commonly extracted from the cell walls of the marine red seaweeds *Gelidium* and *Gracilaria* (103). It is harvested from *Pterocladia, Grasiliaropsis* and *Afeltia* to a lesser extent (104). The use of agar-producing seaweeds in food has been documented since 300 A.D. by the Chinese. In the seventeenth century, the Japanese developed a freeze-thaw technique that enabled the extraction of agar from aqueous extracts. Agar emerged in the 1880's as a culture medium and experienced a boom in its industrial usage due to its ability to form thermally reversible gels (103). It is commonly used as an additive in the food industry for its gelling capabilities, it is produced at nearly 8,000 tons per year as of 2003 (104). As interest in marine polysaccharides for pharmaceutical applications has increased, there has been an increasing appreciation for agar and its derivatives as therapeutics, beyond its rheological properties.

**Figure 6:**
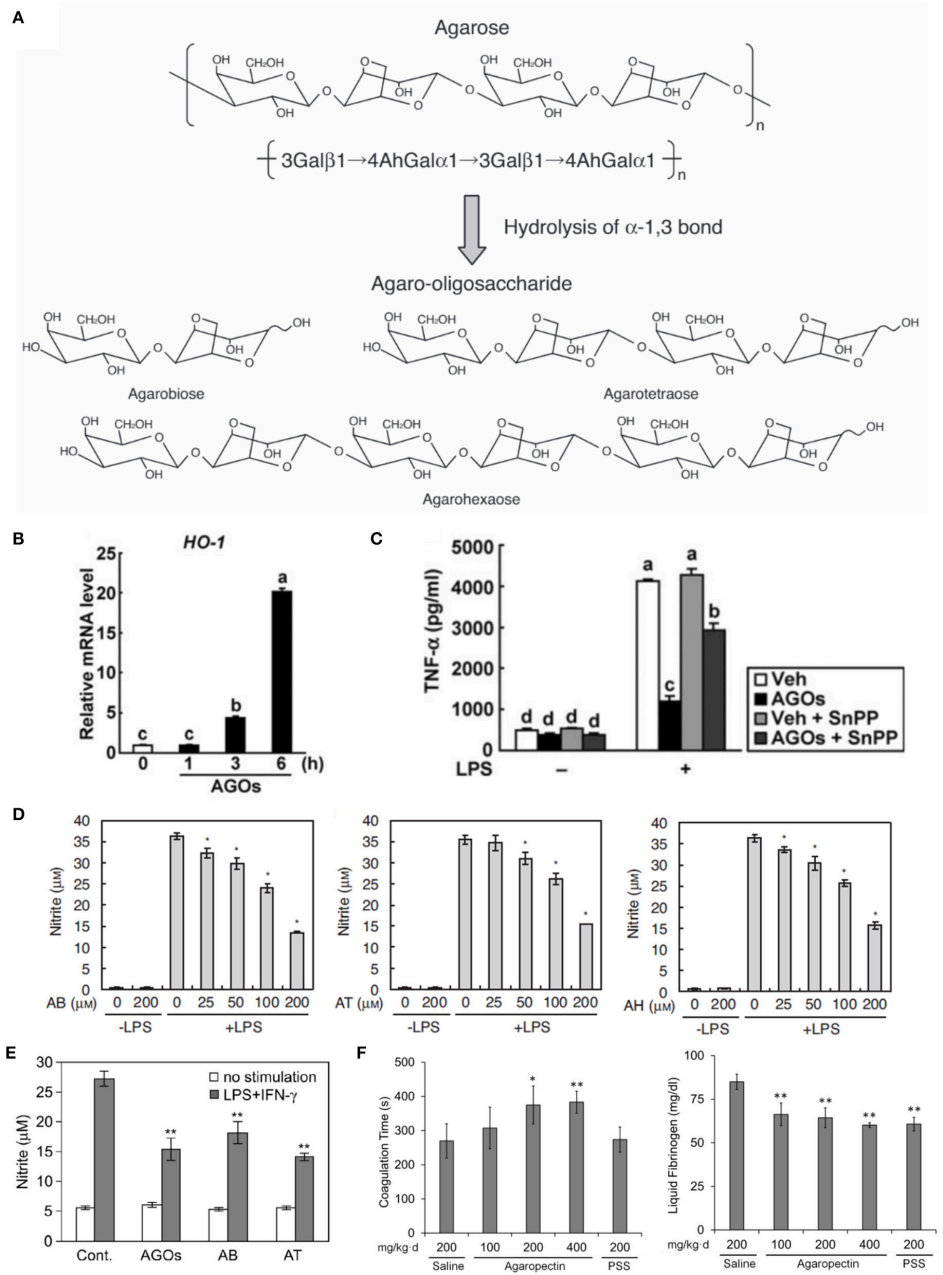
Studies of the effects of agar derivatives on inflammation, oxidative stress, coagulation and bacteria of the gut flora. **(A)** Schematic of agarose hydrolysis yielding agaro-oligosaccharides (AOs) with varying degrees of polymerization (106). **(B)** Heme oxygenase−1 protein levels in the inflammation-induced colons of mice orally administered vehicle (Veh) or AOs (AGOs) (107). **(C)** Harvested mouse colons without colitis induction (sham) or with colitis induction by trinitrobenzene sulfonic acid (TNBS) and oral administration of vehicle (Veh) or AOs (AGOs; left panel). Damage scores for colons (right panel) (107). **(D)** Nitric oxide levels in LPS-stimulated RAW264.7 macrophages treated with the AOs agarobiose (AB), agarotetraose (AT) and agarohexaose (AH). *p* < 0.01 vs. LPS alone (106). **(E)** Nitric oxide production in stimulated peritoneal macrophages from mice orally administered water containing 3% of a mixture of AOs (AGOs), AB or AT at 10 ml/kg per day. ^**^p < 0.01 vs. control with no stimulation (108). **(F)** Coagulation time (left) and plasma fibrinogen concentrations (right) of blood harvested from rats orally administered saline, agaropectin at various concentrations or propylene glycol alginate sulfate (PSS) (109). ^*^p < 0.05 or ^**^p < 0.01 vs. saline.

Agar is comprised of alternating 3, 6-anhydro-L-galactose, and D-galactose units linked by alpha-(1,3) and beta-(1,4) glycosidic bonds (105). Removal of the agaropectin component of agar yields agarose. Coil-double helix transitions of agarose that occur heating and cooling result in the formation of characteristically brittle agar gels and hydrolyzation of agarose by mild acidic conditions produces agar oligosaccharides (AOs) (Figure 6A) (106). Considered the active derivative of agar, AOs are further distinguished by the number of agarobiose units or degrees of polymerization, and the degree of polymerization has been associated with AO physiologic activity (110). Enzymatic hydrolysis of agarose at beta-1,4 glycosidic bonds by beta-agarase produces neoagaro-oligosaccharides (NAOs), which possess D-galactose at the reducing end (106, 111). To date, the effect of AOs on cholesterol metabolism, weight loss, inflammation, oxidative stress, tumor progression and the gut flora has been examined.

Agar has been utilized as a source of water-soluble dietary fiber. As dietary fiber has been observed to influence lipid metabolism, several groups have examined the effects of agar on cholesterol. In a study performed by Ito and Tsuchiya, rats fed casein and sucrose diets supplemented with 1–10% agar for 28 days did not exhibit alterations in fecal cholesterol levels, indicating that agar had no significant effect on cholesterol absorption, owing to the fact that agar does not readily form ionic colloids (112). Total plasma and fecal cholesterol levels in agar-fed rats did not differ significantly from the control diet. Paradoxically, a study conducted by Kiriyama's group found that dietary fiber from agar reduced cholesterol absorption (112, 113). In this study, rats were fed high cholesterol diets for 5–8 days with various algal polysaccharides as sources of dietary fiber. Rats receiving agar absorbed less cholesterol, while exhibiting increased cholesterol levels in the liver, than those fed high cholesterol diets without dietary fiber supplementation (112). Another study examining the effects of algal polysaccharides on lipid levels, performed by Ren and co-workers, observed that agar as a dietary fiber did not significantly affect total cholesterol, HDL or LDL in rats fed a high-cholesterol diet. Systolic blood pressure in these rats also did not differ compared to control rats (no dietary fiber) (63).

Dietary fiber supplementation of agar in humans has also been examined. In a study by Maeda and co-workers, obese Japanese patients with type 2 diabetes and glucose tolerance received 180 g of agar daily for 12 weeks following 4 weeks of a calorie controlled diet. Individuals who received the agar-supplemented diet experienced a greater reduction in glycated hemoglobin (Hb1Ac) compared to those who were not supplemented. Additionally, total cholesterol was significantly reduced in the agar group compared to the control group, while fasting insulin levels were increased. Glucose levels and blood pressure did not significantly differ between groups. The major finding of this study was greater weight loss in the agar-supplemented group (114).

Enoki et al. previously determined that AOs inhibit pro-inflammatory mediator release by inducing heme oxygenase-1 (106). Higashimura et al. expanded on this work, demonstrating that this increase in heme oxygenase-1 expression leads to anti-inflammatory activity in a mouse colitis model. In their study, AOs or vehicle were orally-administered for 3 or6 days at 4 g/kg/day in mice fed a normal diet. Colons harvested from AO-treated mice revealed increased heme oxygenase-1 protein in macrophages in the submucosa (Figure 6B). When colitis was induced via treatment with 100 mg/kg trinitrobenzene sulfonic acid, AO treatment was observed to reduce colonic lesions (Figure 6C) and the activity of tissue-associated myeloperoxidase, an indicator of neutrophil accumulation. Ultimately, their study led to the finding that 3,6-anhydro-L-galatose at the reducing end of AOs is important for promoting the expression of heme oxygenase-1 (107).

Neoagaro-oligosaccharides have been shown to suppress LPS-induced inflammation (111). Wang and co-workers induced macrophage inflammatory responses in RAW264.7 macrophages, examining nitric oxide production following treatment with and without NAOs. NAO pretreatment reduced nitric oxide levels, but neoagarotetraose, neoagarohexaose and neoagarooctaose were efficacious at low doses. Of these three NAOs, neoagarotetraose treatment at 500 μg/ml resulted in nitric oxide levels comparable to the control. Nitric oxide reduction was determined to result from decreased in nitric oxide synthase expression and protein levels in response to NAO treatment. The group also examined the effect of neoagarotetraose on the expression and protein levels of inflammatory cytokines. TNF-α and interleukin-6 protein and mRNA levels were significantly reduced with treatment of neoagarotetraose in a dose-dependent manner. Finally, Wang and co-workers determined that neoagarotetraose inhibits the MAPK and NF-κB signaling pathways, which are involved in macrophage-mediated inflammatory responses (111).

Studies using agar to lower oxidative stress have had mixed results. Chen et al. examined the effect of AOs on the production of ROS, treating human liver L-02 cells for 2 h with 1 mM to 125 μM of either agarobiose, -tetraose, -hexaose, -octaose, and -decaose (110). The 2′,7′-dichlorofluorescin diacetate (DCFH-DA) assay was performed, in which cells were first treated with DCFH-DA followed by either 100 μM of H_2_O_2_ or 25 μM of antimycin A to induce oxidative stress. Fluorescence resulting from the oxidation of DCFH by ROS was measured. The AOs reduced fluorescence in a concentration-dependent manner, with agarohexaose providing the best protection against oxidation. Chen et al. also found that while agarobiose and agarotetraose provided oxidation protection, at lower concentrations, they induced oxidation (110).

Following this work, Enoki and co-workers examined the effects of AOs on nitric oxide production in the RAW264.7 mouse macrophage cell line. The RAW264.7 cells were treated varying concentrations of the AOs agarobiose, agarohexaose or agarotetraose, as well as the NAO neoagarohexaose, and stimulated to produce nitrite via LPS treatment. They observed that AO treatment decreased nitrate production by 50–60% in a dose-dependent manner, with the highest AO doses (200 μM) being the most efficacious (Figure 6D). However, the NAO neo-agarohexaose did not reduce the production of nitric oxide, even at high concentration (1,000 μM). The authors suggested that 3,6-anhydro-galactose at the reducing end of AOs may be necessary for reduction of nitric oxide production, since NAOs with a D-galactose at the reducing end did not alter the production of nitric oxide. This study found no correlation between the degree of polymerization of AOs and efficacy as an antioxidant (106). Enoki and co-workers sought to determine if AOs could reduce nitric oxide production *in vivo* (108). They treated mice orally with water containing 3% agarobiose, agarotetraose or an AO mixture at 10 ml/kg/day for 2 weeks and stimulated macrophage production via RPMI1640 injection into the peritoneal cavity. All three AO treatments reduced nitric oxide production in the harvested macrophages compared to the control group, with agarotetraose producing an approximately 60% reduction in nitric oxide (Figure 6E) (108).

Anticoagulant activity has been described for agaropectin, the other major component of agar. In a study performed by Qi and co-workers, rabbit blood was treated with various concentrations of agaropectin obtained from *Gelidium amansii*, heparin or saline (109). The study found that coagulation time was delayed with increasing concentrations (3.125–25 mg/kg) of agaropectin compared to saline-treated blood. The average coagulation time of blood treated with 25 mg/kg of agaropectin was 670 s, compared to 89.75 s for saline-treated and 690 s for 4 μg/kg heparin-treated blood. The group then examined this activity *in vivo* by orally administering agaropectin to rats. The rats received 100, 200, and 400 mg/kg of agaropectin, saline (negative control) or propylene glycol alginate sulfate (PSS, positive control) daily for 15 days and later sacrificed for blood harvest. Coagulation time, thrombin time, prothrombin time and activated partial thromboplastin time were all increased for the agaropectin-treated groups in a dose-dependent manner (Figure 6F). The 400 mg/kg/day agaropectin dose produced the largest increases for those times, and plasma fibrinogen levels for that group also reduced significantly compared to the positive control group (Figure 6F). Further studies are required to confirm whether agaropectin induces fibrinolysis directly (109).

There has been increasing interest in dietary prebiotics, typically fibrous compounds consumed with the intention of supporting the activity of beneficial gut bacteria. Both AOs and NAOs have been shown to have such prebiotic effects (105, 115, 116). The efficacy of AOs and NAOs as prebiotics has been associated with degrees of polymerization, as prebiotic activity has been observed to decrease with the progressive breakdown of AOs and NAOs (105, 115). Gut metagenome studies have shown that agarases occur frequently in the Japanese population, and may be evidence of gene transfer between gut bacteria and environmental bacteria associated with food (117).

Li et al. determined that *Bacteroides uniformis* L8, a gut bacterium isolated from Chinese subjects, is capable of hydrolyzing agarose and AOs obtained from agarose, producing intermediates that can utilized by the beneficial *Bifidobacterium adolescentis* and *Bifidobacterium infantis*. Furthermore, the final product of this hydrolysis, D-galactose, can be utilized by the B2 strain of *E. coli*. Fermentation of AOs by *B. uniformis* L8 and *E. coli* B2 together resulted in near complete utilization of AOs (105). The authors noted that in a study performed by Cano and co-workers, *B. uniformis* CECT7771 provided metabolism regulation and some protection from infection in obese mice, (118) suggesting that *B. uniformis* L8 should be explored for similar potential. A role for AOs in promoting such beneficial bacteria merits additional exploration. The abundance of agar in the food industry makes it relatively inexpensive to access and lowers barriers to its entry as a therapeutic. Its derivatives have attracted attention for their ability to reduce inflammation, lower oxidative stress and delay coagulation *in vivo*. As a prebiotic, agar may have the potential to alleviate complex disorders related to the gut.

## Carrageenan

Carrageenan is part of the galactan family of sulfated polysaccharides and is commonly extracted from red seaweed that grows in the Atlantic Ocean. Galactans have been studied for their effects on atherosclerosis, lipid lowering, and blood coagulation (119, 120). Carrageenan has been employed in multiple ways since its first adoption as a gelatin and cough remedy in 400 A.D. Ireland. Today, the seaweed extract is commonly used in food processing and pharmaceutical testing (121). Structurally, carrageenan is a sulfated polygalactan that comprises of roughly 15–40% ester-sulfate groups. Alternating units of D-galactose and 3,6-anhydro-galactose form the main structure of carrageenan along with α-1,3- and β-1,4-glycosidic linkages. Both the position and number of sulfate groups comprising the structure dictate the activity of carrageenan. Designations such as λ, κ, ι, ε, and μ are commonly used to distinguish between varying sulfate group characteristics (121). As observed carrageenan subtypes have increased, a binomial system has been implemented for further distinction (122). To succinctly convey the position of groups, G and D refer to the galactose groups in a single binomial unit while DA refers to anhydro-galactose and #S denotes the number of sulfate groups. For example, κ carrageenan could be denoted as G4S DA. Three of the most well researched forms of carrageenan are kappa (κ), iota (ι), and lambda (λ), corresponding to one, two or three sulfate groups on the galactose backbone, respectively. The gelling and thickening effects of these forms of carrageenan have been extensively used in food processing. Iota and κ forms are used to create gels while the λ form cannot gel and is used instead as a thickener (123, 124)

Murata et al. conducted multiple animal studies to test the effect of seaweed-derived compounds on atherosclerotic development (125, 126). Rabbits were fed a high cholesterol diet and given daily intravenous injections of treatments including carrageenan for 18 weeks. Gross and microscopic analyses of harvested aortic and coronary artery tissue were performed after the 18-week period. Atherosclerotic lesions were graded on a scale of 0–5, with 0 representing no lesion formation and 5 being severe lesion formation with intraluminal protuberance. The saline control group exhibited a mean score of 3.84 while the carrageenan group had a mean score of 1.88 (125). In a second similar experiment by Murata et al, the control scored an average of 4.01 while the carrageen treated group averaged 1.44. Histological analysis confirmed that lesions were more commonly found in the control group compared to the carrageenan treated rabbits (126).

**Figure 7:**
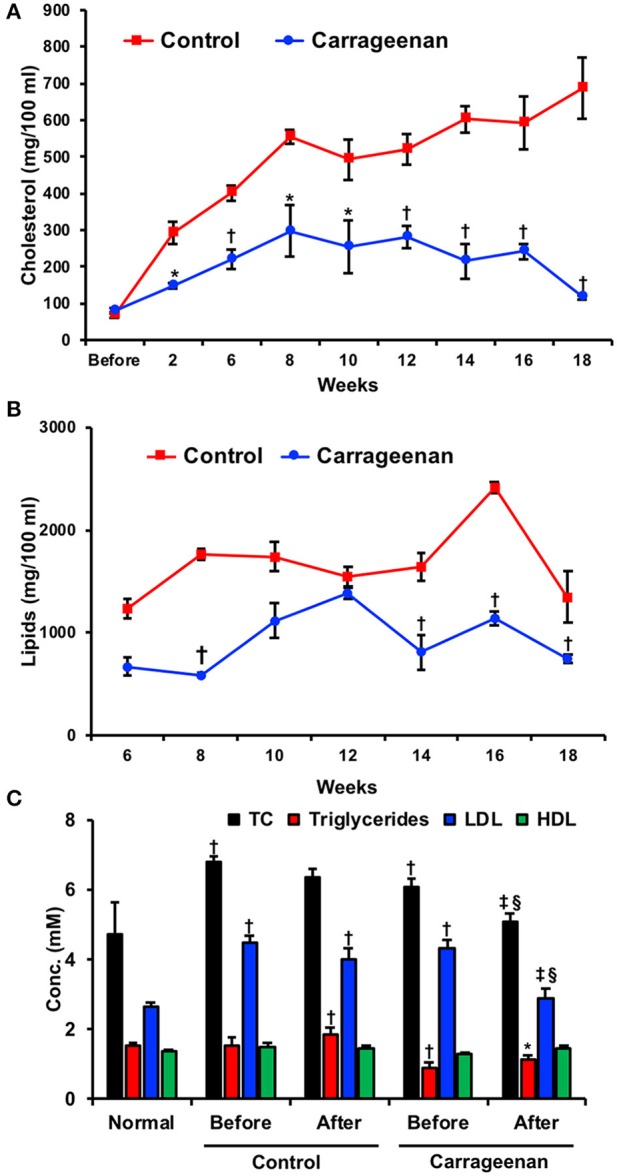
Lipid lowering activity of carrageenan. Rabbits were fed a high cholesterol diet and given daily 5 mg via intravenous injections of treatments including carrageenan for 18 weeks. **(A)** Lipid and **(B)** cholesterol serum levels were measured before and every two weeks after the start of the diet. A carrageenan-enriched diet demonstrated lower readings of both lipids and cholesterol at multiple time points. ^*^p < 0.05 or ^†^*p* < 0.01 vs. control group. Used with permission (125). **(C)** Twenty human volunteers were given a carrageenan-enriched diet and monitored for 8 weeks. Groups included a healthy control group and an experimental group with ischemic heart disease. Both groups experienced reductions in total cholesterol (TC) and HDL. ^*^Designates significantly different from all conditions (*p* < 0.05). ^†^Significantly different compared to control group (*p* < 0.05).^‡^Significant difference between “before” and “after” groups in either the control or experimental groups (*p* < 0.05). ^§^Significantly different between control “after” group and experimental “after” group (*p* < 0.05). Used with permission (127).

Carrageenan has also been extensively studied for its ability to lower lipid levels. This characteristic was originally recognized in the 1960s by Murata et al through the aforementioned rabbit studies (125, 126). Rabbits were fed a high cholesterol diet and given daily intravenous injections of treatments including carrageenan for 18 weeks. Lipid and cholesterol serum levels were measured through blood samples drawn every 2 weeks (Figures 7A,B). Treatment with carrageenan showed significantly lower cholesterol, lipid, and phospholipid serum levels from the beginning of the study until the end at week 18 compared to the saline control. The results of this study were replicated by Murata in a second publication a year later, which confirmed carrageenan's lipid lowering effect. The lipid lowering activity of carrageenan was also observed by Ito and Tsuchiya using rats fed a supplement of cholesterol for 28 days. Here also, carrageenan significantly reduced cholesterol levels in the plasma (128).

This activity was then studied in humans, in an experiment where 20 volunteers consumed carrageenan-containing food for 8 weeks (127). Serum cholesterol and triglyceride levels were measured (Figure 7C). Total cholesterol levels at the end of the study were significantly lowered for those consuming carrageenan-containing foods (3.64 ± 1.43 mmol/L) compared to those consuming their usual diet (5.44 ± 1.98 mmol/L). The mean difference for total cholesterol levels between the groups was 1.80 mmol/L or 33%. Triglyceride measurements followed a similar pattern with a mean difference of 1.20 mmol/L or 32% between the carrageenan and control groups. HDL cholesterol levels were found to increase in the carrageenan group compared to the control with a mean difference of 0.40 mmol/L or 32%. LDL cholesterol levels were not significantly changed.

Due to its high sulfate content, carrageenan's effects on coagulation have been studied. Murata et al. hypothesized that highly sulfated carrageenan groups could act like heparin sulfate and be a potent anti-coagulant (125, 126). However, they could not find a conclusive link between carrageen and coagulation. Carrageenan has been suggested to have an anti-thrombin effect and disrupt platelet aggregation. More recent studies have been able to further explore this hypothesis *in vivo* (123, 129–132). Silva et al. tested the effects of λ, ι, and κ carrageenan on coagulation using an aPTT assay (123). This technique measures the activity of the coagulation cascade by measuring the time that it takes for a clot to form *in vitro*. The aPTT assay revealed that the least sulfate containing carrageenan types, κ and ι, scored aPTTs of 132.2 and 240 s, respectively with 100 μg treatments. The most sulfated group, λ, achieved an aPTT of 240 s with 20 μg of treatment, strengthening the initial hypothesis by Murata et al. However, each of these forms of carrageenan is significantly outperformed by heparin sulfate with a tested aPTT of 250 s with 2.5 μg of treatment. Some *in vivo* work has been performed in rabbits where Schimpf et al. observed an anti-thrombin effect in rabbit plasma that leads to disruption of platelet aggregation (133).

While the above effects of carrageenan can be seen as beneficial in many disease states, the seaweed derivative has also been shown to induce acute inflammation. Many animal models that test anti-inflammatory treatments are centered on the ability of carrageenan to induce local inflammation. Carrageenan has been used as a proinflammatory agent in paw and ear edema models as well as in the lungs to induce pleurisy (123). Unlike other modes of inflammation, histamine and 5-hydroxytryptamine do not play a part in carrageenan-induced inflammation (7, 134). Di Rosa et al. further examined the mechanism behind carrageenan's proinflammatory effect (135). They discovered that carrageenan has the ability to induce the release of kinin-like compounds which can act as mediators to inflammation (136). Upon treatment with cellulose sulfate, which has been shown to deplete kininogen activity, carrageenan-induced edema was suppressed in rats (137). These experiments gives credence to the authors' hypothesis that increased kinin production could be an underlying mechanism behind the inflammation response but much is still unknown regarding kinin production and activity.

More recently, Silva et al. compared the proinflammatory effects of the different forms of carrageenan (123). They observed both paw edema as well as pleurisy in rats that had been injected with κ, ι, and λ carrageenan. Carrageenan concentrations ranged from 0.1 to 1%. Inflammation varied significantly among the 1% groups with κ, ι, and λ carrageenan increasing paw size by an average of 3.7, 4.0, and 4.2 mm, respectively, compared to the saline contralateral control group. Injection of carrageenan into the plural cavity elicited increased polymorphonuclear cells and nitric oxide production. All carrageenan groups showed significantly greater levels of each parameter compared to saline. λ carrageenan showed the largest number of polymorphonuclear cells while ι produced the most nitric oxide. Overall, ι carrageenan was concluded to stimulate the most significant proinflammatory effect. While the potency of carrageenan is normally associated with the number or sulfate groups, ι carrageenan has neither the highest nor lowest sulfate content, making this an intriguing result. This study helps to illustrate the importance of carrageenan selection in the testing of anti-inflammatory treatments. Overall, carrageenan's capacity to decrease lipid levels has been continually supported through multiple publications, but its use as an inflammatory agent has become a standard model in anti-inflammatory research. This scenario makes it a complex candidate for atherosclerosis treatment. Fewer circulating lipids would presumably lessen the rate at which plagues form, but inflammation could initiate endothelial dysfunction, adhesion molecule activation, and the other downstream effects of atherosclerosis. Despite being the focus of research spanning more than 50 years, carrageenan continues to remain a contentious potential treatment for atherosclerotic plaque formation.

## Conclusions

Many seaweed derivatives appear to have promising properties for reducing atherosclerosis and disease processes associated with atherosclerosis. However, there seems to be no general correlation between the various structures of the polysaccharides, such as length of chain and sulfation, and their anti-atherogenic potential. Notably, a higher degree of sulfation generally led to higher anti-coagulant activity, although agar, a non-sulfated polysaccharide, also inhibited coagulation. Laminarin appeared to have a greater effect on atherosclerosis risk factors in its sulfated form, possibly to its increased resemblance to the endogenous heparan sulfates or heparin. Of the polysaccharides discussed, only fucoidan, laminarin sulfate and carrageenan were shown to directly hinder atherosclerotic lesion development. Others, such as alginate, ulvan and agar, lowered risk factors involved in the progression of atherosclerosis but there is no direct evidence that they can reduce atherosclerotic lesion progression. Since both alginate and ulvan have sulfated forms, the anti-atherogenic effect perhaps depends on the sugars in these polysaccharides and their similarity to endogenous compounds rather than sulfation. However, further research is needed to confirm the mechanisms of action of marine polysaccharides and form a conclusive link between chemical structures and the end effect on atherosclerosis. There has been limited mechanistic investigation of the properties seaweed polysaccharides on atherosclerosis. Extensive studies were performed on heparin to discover the importance of sulfation patterns, molecular weight and chemical modifications on its anti-coagulant activities. Similar studies are needed with regard to the activities of complex algal polysaccharides with potential in treating atherosclerosis. These studies have the potential to reveal highly active forms of the polysaccharides that can have increased efficacy.

Polysaccharides derived from seaweeds have many practical aspects that make them appealing therapeutics for chronic disease. They are widely available in large quantities and centuries of human consumption support their safety as part of the diet. From a regulatory perspective, these natural products have challenges including the inherent heterogeneity of the material derived from seaweeds, which depends on the type of seaweed, season and many other factors that are readily controllable from seaweeds harvested from the ocean. However, other natural polysaccharides have been widely used including heparin, which is derived from porcine or bovine tissue. Thus, there is some precedent for using these types of natural products, albeit with the caveat that characterization is needed to provide uniformity of the sample and that contamination can occur with other polysaccharides that may have negative effects. As the polysaccharides may have a similar structure it is not as apparent as it may be with other drug types, with the notable example of contamination of heparin with over-sulfated chondroitin sulfate, which lead to 81 deaths patients in 2008. There are activities in common for many of the sulfated polysaccharides, including anti-coagulant activity, lipid lowering and heparin-like activity. The ability of seaweed polysaccharides to shift the gut microbiome is a relatively unexplored activity and merits further study for other types of seaweeds and in human patients. Overall, there is great therapeutic potential in seaweed-derived polysaccharides with the added benefits of low cost, abundant supply and low toxicity that enables chronic dietary use. These aspects make them highly appealing as a counter to the chronic nature and massive prevalence of atherosclerotic disease.

## Author contributions

All authors listed have made a substantial, direct and intellectual contribution to the work, and approved it for publication.

### Conflict of interest statement

The authors declare that the research was conducted in the absence of any commercial or financial relationships that could be construed as a potential conflict of interest.

## Footnotes

^1^http://www.aphotomarine.com/
